# Dissimilar Impact of a Mediterranean Diet and Physical Activity on Anthropometric Indices: A Cross-Sectional Study from the ILERVAS Project

**DOI:** 10.3390/nu11061359

**Published:** 2019-06-17

**Authors:** Marta Sánchez, Enric Sánchez, Marta Hernández, Jessica González, Francesc Purroy, Ferran Rius, Reinald Pamplona, Cristina Farràs-Sallés, Liliana Gutiérrez-Carrasquilla, Elvira Fernández, Marcelino Bermúdez-López, Javier Salvador, Jordi Salas-Salvadó, Albert Lecube, on behalf of the ILERVAS project collaborators

**Affiliations:** 1 Endocrinology and Nutrition Department, Arnau de Vilanova University Hospital, Obesity, Diabetes and Metabolism (ODIM) Research Group, IRBLleida, University of Lleida, 25198 Lleida, Spain; ma.san.pe.88@gmail.com (M.S.); esanchez@irblleida.cat (E.S.); martahernandezg@gmail.com (M.H.); frius.lleida.ics@gencat.cat (F.R.); liligutierrezc@gmail.com (L.G.-C.); 2 Respiratory Department, Arnau de Vilanova-Santa María University Hospital, Translational Research in Respiratory Medicine, IRBLleida, University of Lleida, 25198 Lleida, Spain; jegonzalez.lleida.ics@gencat.cat; 3 Centro de Investigación Biomédica en Red de Enfermedades Respiratorias (CIBERES), Instituto de Salud Carlos III (ISCIII), 28029 Madrid, Spain; 4 Stroke Unit, Arnau de Vilanova University Hospital, Clinical Neurosciences Group, IRBLleida, University of Lleida, 25198 Lleida, Spain; fpurroygarcia@gmail.com; 5 Department of Experimental Medicina, IRBLleida, University of Lleida, 25198 Lleida, Spain; reinald.pamplona@mex.udl.cat; 6 Applied Epidemiology Research Group, IRB Lleida, 25198 Lleida, Spain; cfarras.lleida.ics@gencat.cat; 7 Unitat de Suport a la Recerca Lleida. Fundació Institut Universitari per a la recerca a l’Atenció Primària de Salut Jordi Gol i Gurina (IDIAPJGol), 08006 Barcelona, Spain; 8 Vascular and Renal Translational Research Group, IRBLleida, RedinRen-ISCIII, 25198 Lleida, Spain; elvirafgiraldez@gmail.com (E.F.); mbermudez@irblleida.cat (M.B.-L.); 9 Endocrinology and Nutrition Department, Clínica Universidad de Navarra, 31008 Pamplona, Spain; jsalvador@unav.es; 10 Human Nutrition Unit, Biochemistry and Biotechnology Department, University Hospital of Sant Joan de Reus, IISPV, Rovira i Virgili University, 43201 Reus, Spain; jordi.salas@urv.cat; 11 Centro de Investigación Biomédica en Red de Fisiopatología de la Obesidad y Nutrición (CIBEROBN), Instituto de Salud Carlos III (ISCIII), 28029 Madrid, Spain; 12 Centro de Investigación Biomédica en Red de Diabetes y Enfermedades Metabólicas Asociadas (CIBERDEM), Instituto de Salud Carlos III (ISCIII), 28029 Madrid, Spain

**Keywords:** adiposity, body fat, Mediterranean diet, obesity indices, physical activity, questionnaire

## Abstract

There is a close relationship between lifestyle behaviors and excess adiposity. Although body mass index (BMI) is the most used approach to estimate excess weight, other anthropometric indices have been developed to measure total body and abdominal adiposity. However, little is known about the impact of physical activity and adherence to a Mediterranean diet on these indices. Here we report the results of a cross-sectional study with 6672 middle-aged subjects with low to moderate cardiovascular risk from the Ilerda Vascular (ILERVAS) project. The participants’ adherence to physical activity (International Physical Activity Questionnaire short form) and MedDiet (Mediterranean Diet Adherence Screener) was evaluated. Measures of total adiposity (BMI, Clínica Universidad de Navarra-Body Adiposity Estimator (CUN-BAE), and Deurenberg’s formula), central adiposity (waist and neck circumferences, conicity index, waist to height ratio, Bonora’s equation, A body adiposity index, and body roundness index), and lean body mass (Hume formula) were assessed. Irrespective of sex, lower indices of physical activity were associated with higher values of total body fat and central adiposity. This result was constant regardless of the indices used to estimate adiposity. However, the association between MedDiet and obesity indices was much less marked and more dependent on sex than that observed for physical activity. Lean body mass was influenced by neither physical activity nor MedDiet adherence. No joint effect between physical activity and MedDiet to lower estimated total or central adiposity indices was shown. In conclusion, physical activity is related to lower obesity indices in a large cohort of middle-aged subjects. MedDiet showed a slight impact on estimated anthropometric indices, with no joint effect when considering both lifestyle variables. ClinTrials.gov Identifier: NCT03228459.

## 1. Introduction

Obesity trends are increasing progressively and are especially influenced by changes in the lifestyle behaviors, especially a decrease of total physical activity and increase of caloric intake [1,2]. Obesity’s growing prevalence is associated with a high social and economic impact, and according to the World Health Organization, there are more than 2.8 million annual deaths associated with diseases related to excess body weight [3]. In fact, adiposity excess has been linked with a wide range of diseases, including cardiovascular (CV) disease, cancer, type 2 diabetes, and sleep apnea-hypopnea syndrome [4]. Positively, even in moderate interventions, changes towards healthy habits—such as balanced diet and regular physical activity—have been reported to decrease body fat mass, improve lean body mass, and reduce overweight and obesity [5,6].

The traditional dietary pattern from populations bordering the Mediterranean Sea is characterized by an abundant consumption of extra-virgin olive oil, vegetables, fruits, legumes, and nuts and moderate consumption of fish and seafood, fermented dairy products, poultry, and red wine [7,8]. This pattern, the so-called Mediterranean diet (MedDiet), exerts potential benefits for certain types of cancer, type 2 diabetes, and some neurodegenerative diseases [9,10,11] and also prevents mortality from cardiovascular diseases [12,13]. MedDiet has been associated with lower total and regional adiposity in reproductive-aged women as well as with lower visceral and subcutaneous abdominal fat tissue in the Italian population [14,15]. Also, Barrea et al. have recently reported a novel association between the adherence to the MedDiet and phase angle, a direct measure by bioelectrical impedance analysis (BIA) used as a marker of cell membrane integrity and predictor of morbidity and survival in many diseases [16]. However, less is known about the role of MedDiet on total body fat and abdominal adiposity. On the other hand, physical activity practice also reduced body fat in middle-aged subjects, showing to be a stronger contributor to the variability in body fat than dietary habits [5,6]. Nevertheless, the associative and synergistic impact of the MedDiet and physical activity on anthropometric indices related to body composition has not been previously evaluated.

In clinical and epidemiological studies, body mass index (BMI) is widely used to assess the presence of obesity. However, BMI classification is far from perfect for providing an accurate measurement of body fat amount and distribution [17,18]. However, the gold standard techniques to directly assess total adipose tissue and abdominal adiposity, such as dual-energy x-ray absorptiometry (DXA) and magnetic resonance imaging, still show a limited widespread in clinical practice, largely because of their complexity, high cost, and time-consuming protocols [19]. For this reason, several mathematical indices that combine traditional anthropometric measures have been developed to estimate total body fat and abdominal adiposity [20,21,22,23,24,25,26]. However, little is known regarding the impact of MedDiet and physical activity on these estimated measurements.

On this basis, the main objective of this study was to appraise the associations between adherence to the MedDiet and intensity of physical activity practice, as well as their simultaneous influence on adiposity estimated by different indices in a large cohort of middle-aged overweight participants. For this purpose, we applied two validated questionnaires, three indices of total adiposity, seven central adiposity measurements, and one lean body mass formula. The study was done according to sex, as its disparity of adiposity distribution and body fatness has been well documented [27].

## 2. Materials and Methods

### 2.1. Design of the Study and Description of the Study Population 

A total of 6672 subjects were enrolled between 2015 and 2017 from 29 primary health care centers in the ongoing Ilerda Vascular (ILERVAS) study dealing with the subclinical atheromatous disease in Lleida, Catalonia, Spain (ClinTrials.gov Identifier: NCT03228459) [28]. Participants with an open history in the electronic primary care medical history database (e-CAP) were selected through simple randomization without replacement, within clusters defined by basic healthcare area. The participant selection process is shown in Supplemental Figure S1. The inclusion criteria were as follows: men and women between 45 and 70 years with at least one cardiovascular risk factor (hypertension, smoking, obesity, dyslipidemia, or having a first-degree relative with premature cardiovascular disease). The exclusion criteria were the presence of any type of diabetes, chronic kidney disease, cardiovascular disease (angina, myocardial infarction, stroke, peripheral artery disease, heart failure, history of vascular surgery), active neoplasia, institutionalized population, a life expectancy of less than 18 months, and pregnancy. A severe functional limitation or a significant cognitive deterioration were not considered exclusion criteria except in the case that they prevented the participants from reaching the place where the anthropometric measures were performed or responding adequately to the administered questionnaires. The prescribed antihypertensive, lipid-lowering, and antithrombotic treatments were acquired from prescription- and pharmacy-invoicing databases provided by the Catalan Health Service, which are incorporated yearly into the information system for the development of research in primary care database.

### 2.2. Adherence to the Mediterranean Diet Assessment

To quantitatively assess the adherence to the MedDiet, the validated 14-item Mediterranean Diet Adherence Screener (MEDAS) was used. This questionnaire was developed to rapidly control the compliance with the dietary intervention in the *Prevención con Dieta Mediterránea* (PREDIMED) *trial* [19,29]. The frequencies of consumption of olive oil, wine, fruits, vegetables, fish, legumes, sofrito (a Mediterranean sauce made with virgin olive oil, tomato, onions, garlic, and species), and nuts were evaluated. The intake of meat or meat-products, butter, bakery products, and sweetened soft beverages per week was also involved in the composite score. Information was obtained through self-reported data, and the final score ranged from 0 to 14 and classified participants according to their level of adherence to MedDiet: high (score ≥11 points), moderate (7–10 points), and low (≤6 points) adherence [11].

### 2.3. Physical Activity Intensity and Type Assessment 

The short version of The International Physical Activity Questionnaire (IPAQ) was administered to all participants. This questionnaire was developed to evaluate physical activity and inactivity in adults [30]. The IPAQ short form asks questions about three detailed types of activity undertaken in four domains: leisure time, domestic and gardening, work-related, and transport-related physical activity. The detailed types of activity that were assessed were walking, moderate-intensity, and vigorous-intensity actions. The metabolic equivalent of task (METs)-min per week, a multiple of the resting metabolic rate, was assessed. Participants were classified following the IPAQ procedure into vigorous, moderate, and low physical activity [30]. The vigorous group was defined as those subjects with vigorous intensity activity on ≥3 days achieving a minimum total physical activity of at least 1500 MET-min per week, or ≥7 days of any combination of physical activity achieving ≥3000 MET-min per week. Those who scored moderately on the IPAQ engaged in ≥3 days of vigorous intensity activity and/or walking of at least 30 min per day, or ≥5 days of moderate intensity activity and/or walking of at least 30 min per day, or ≥5 days of any combination achieving ≥600 METs min per week. Those participants with any of the criteria for either moderate or high levels of physical activity were classified as the low level of physical activity group [30].

### 2.4. Assessment of Obesity-Related Parameters

Weight and height were measured in light clothing and without shoes using standard equipment, to the nearest 0.5 kg and 1.0 cm, respectively. BMI was calculated as the ratio of body weight (kg) to the height (m) squared [31]. A non-stretchable tape with an accuracy of 0.1 cm was used to evaluate waist and neck circumferences. Waist circumference was measured midway between the lowest rib and the iliac crest on the horizontal plane with the subject in a standing position [32]. Neck circumference was measured in a plane as horizontal as possible, immediately below the laryngeal prominence, while standing erect with eyes facing forward [33]. All measurements were performed under strictly standardized conditions by one experienced nurse, using the same device to avoid inter-observer and inter-device variability. The relative intra-evaluator technical error measurement was lower than 1% for height, weight, waist, and neck circumferences. As the hip circumference was not measured in our population, anthropometric indices, including hip circumference, were not considered in our study.

Two equations were added to BMI to estimate total body fat: the Clínica Universidad de Navarra-Body Adiposity Estimator (CUN-BAE) and the formula proposed by Deurenberg et al. [20,21]. The CUN-BAE was calculated as follow: −44.988 + (0.503 × age) + (10.689 × sex) + (3.172 × BMI) − (0.026 × BMI^2^) + (0.181 × BMI × sex) − (0.02 × BMI × age) − (0.005 × BMI^2^ × sex) + (0.00021 × BMI^2^ × age), where male is 0 and female is 1 for sex, and age in years. The Deurenberg equation assessed body fat using the formula: (1.20 × BMI) + (0.23 × age) − (10.8 × sex) − 5.4, where sex is 0 for females and 1 for males.

Five equations were added to the waist and neck circumferences to estimate central adiposity: (i) the conicity index proposed by Valdez et al. in 1991, (ii) the waist to height ratio, (iii) the Bonora’s equation, (iv) the A body adiposity index developed by Krakauer et al. in 2012, and (v) the body roundness index proposed more recently by Thomas et al. [22,23,24,25,26]. The conicity index is based on the hypothesis that people that accumulate abdominal fat have a silhouette like a double cone (that is, two cones sharing the same base, one positioned over the other), whereas subjects with less visceral fat have the shape of a cylinder [22]. Therefore, conicity index ranges from 1.73 (a perfect double cone) to 1.0 (a perfect cylinder) and was obtained following the next equation: 0.109^−1^ × waist circumference (m) × (weight (kg)/height (m))^−1/2^ [22].

The waist to height ratio was calculated as waist circumference (m) divided by height (m) [23]. The equation proposed by Bonora et al. uses different formulas according to gender: −453.7 + (6.37 × waist circumference) for men, and −370.5 + (4.04 × waist circumference) + (2.62 × age) for women [24]. To obtain the A body shape index, the eccentricity of the body (ε) is determined first [25]. The ε is a non-dimensional value that quantifies the degree of circularity of an ellipse, ranges from zero (perfect circle) to one (a vertical line) and is calculated by the formula: (1 − π ^−2^ × waist circumference (m)^2^ × height (m) ^−2^)^1/2^. Subsequently, the next formula calculates the index: 364.2 – (365.5 × ε), in which values closer to 1 are related to rounder individuals, whereas lower values are associated with leaner individuals [25]. Finally, the body roundness index was calculated as: waist circumference (m)/(BMI^2/3^ × height (m)^1/2^) [26].

Finally, the Hume formula was applied to estimate lean body mass: 0.32810 × weight (kg) + 0.33929 × height (cm) − 29.5336 (for males), and 0.29569 × weight (kg) + 0.41813 × height (cm) − 43.2933 (for females) [34].

### 2.5. Covariates Assessment

The smoking status (never/current/former) was recorded. Smokers who stopped smoking ≥1 year prior to recruitment were considered former smokers. Blood pressure was measured in triplicate, after 5 min of rest using an automated device (Omron M6 Comfort HEM-7221-E (Omron Healthcare, Kyoto, Japan)) at 2-min intervals, and the mean of the last two was calculated. A dried capillary blood sample was used for the total cholesterol (mg/dL) measurement using a REFLOTRON^®^ (Roche Diagnostics, GmbH, Germany).

### 2.6. Ethical Approval

Informed consent was obtained from all subjects involved in the study. The procedure was approved by the Arnau de Vilanova University Hospital ethics committee (CEIC-1410). Moreover, the study was also completed according to the ethical guidelines of the Helsinki Declaration and Spanish regulation concerning the protection of personal data.

### 2.7. Statistical Analysis

Subjects were classified based on the adherence to both MedDiet (low/moderate/high) and physical activity practice (low/moderate/vigorous). The normal distribution of the variables was evaluated using the Shapiro-Wilk test. Given its skewed distribution, quantitative data were expressed as the median [interquartile range]. Baseline characteristics across categories were compared by the Kruskal-Wallis test (with a Bonferroni post-hoc analysis) and the Pearson’s Chi-squared test for continuous and categorical variables, respectively. Also, two-way ANOVA was used to determine how obesity indices were affected by the adherence to both physical activity and MedDiet together. *p*-values <0.05 were considered statistically significant. All statistical tests were done using the SSPS statistical package (IBM SPSS Statistics for Windows, Version 25.0. Armonk, NY, USA).

## 3. Results

Most participants in the ILERVAS project showed a low physical activity practice (66.0%) and a moderate adherence to MedDiet (75.7%) (Table 1 and Table 2). Subjects with low physical activity practice were mainly men, older, with a higher BMI, as well as a higher prevalence of hypertension and hypercholesterolemia than participants who referred to practice a vigorous physical activity (Table 1). In both genders, lower indices of physical activity were associated with higher values of total body fat and central adiposity (Table 3). This result was constant regardless of the indices used to estimate adiposity. However, physical activity practice was not related to lean body mass estimated by the Hume formula.

Participants with lower adherence to MedDiet were mainly men, younger, with a higher BMI, and a higher prevalence of smoking history than subjects who referred to higher adherence to diet (Table 2). Curiously, the lower adherence to MedDiet was also associated with a lower prevalence of hypertension and dyslipidemia. The association between adherence to MedDiet and obesity indices was much less marked and more dependent on sex than that observed when analyzing physical activity (Table 4). Male participants with higher adherence to MedDiet showed lower values of central adiposity (conicity index, waist to height ratio, A body shape index, and body roundness index) than participants with moderate adherence (*p* ≤ 0.036 for all), without differences in total adiposity indices. Contrary, female participants with high adherence to MedDiet presented lower total (BMI and CUN-BAE) and central adiposity (waist to hip ratio and body roundness index) measurements in comparison to those females with moderate adherence to MedDiet.

Finally, Table 5 exhibits the interactions between physical activity and MedDiet adherence with adiposity indices: a positive association was detected in both genders, but not observed when adherence of MedDiet was included in the joint analysis.

## 4. Discussion and Conclusions

The present study compares the impact of physical activity and adherence to MedDiet on different equations used to estimate total body fat and abdominal adiposity in a middle-aged and overweight Caucasian cohort. Our data support the concept that physical activity is related to low adiposity in both genders, showing a positive association in almost all of the assessed indices. However, and contrary to expected, the ability of the MedDiet to modulate the amount of total and central adiposity was small in magnitude. Also, in our population, it is noteworthy that there was no association between combined physical activity and MedDiet adherence and obesity indices.

Our results, together with the continuing worldwide increase in body weight and the favorable impact of regular exercise for reducing the morbidity and mortality associated with cardiovascular disease and diabetes, suggest that the physical activity is a critical behavior among lifestyle strategies, aiming to prevent excess weight and reduce fat mass in overweight subjects [35,36]. In the Look AHEAD trial, among 2240 subjects with type 2 diabetes and obesity, lower physical activity measured with an accelerometry was associated with higher values of BMI [37]. Recently, in a senior Spanish cohort at high cardiovascular disease, greater time spent on moderate to vigorous physical activity and fewer on sedentary behaviors was inversely associated with the prevalence of obesity and some components of the metabolic syndrome [38]. Similarly, in the 2003–2006 National Health and Nutrition Examination Survey, the moderate-to-vigorous physical activity assessed by accelerometers was strongly and negatively associated with BMI and waist circumference, but also with triceps and subscapular skinfolds [39]. However, data about the impact of physical activity on other parameters of total body fat or abdominal adiposity are scarce. Another cross-sectional study of 1009 men aged 71–91 years from primary care centers in the UK showed that each daily additional 30 min of moderate-to-vigorous physical activity was associated with a 0.60 reduction in the fat mass index [40]. More recently, Cameron et al. have demonstrated that moderate-to-vigorous physical activity was negatively associated with percent body fatness and visceral adipose tissue assessed by DXA in 298 overweight adults [41]. In this study, the inverse relationship between physical activity and percentage of body fat was stronger for non-Latinos than for Latinos, a fact that introduces the possibility that differences in diet and eating habits might modulate the impact of physical activity on anthropometric indices [41]. The positive impact of physical activity on weight and abdominal fat reduction also appears without dietary restriction [42,43].

The underlying mechanisms driving the positive impact of physical activity on anthropometric indices are poorly understood, but they might involve the higher sensitivity to lipolytic stimulation and less to insulin suppression of visceral adipose tissue relative to subcutaneous fat [44,45]. Also, skeletal muscles secrete interleukin (IL)-6 into the circulatory system during intensive exercise, an interleukin that acts as both a pro-inflammatory cytokine and an anti-inflammatory myokine, which has also been identified as a lipolytic agent [46,47]. Finally, the role of irisin, a novel myokine secreted in response to physical activity, needs to be considered. Irisin exerts its major action by increasing the expression of mitochondrial uncoupling protein 1, which facilitates the conversion of white adipose tissue into beige adipose tissue [48]. Under resting conditions, serum irisin concentrations do not relate to body fat or lean body mass assessed by DXA in healthy humans [49]. However, 8-week endurance-training intervention increased circulating irisin levels in middle-aged adults, and this change occurred concomitantly with reductions in whole-body fat mass and abdominal visceral adipose tissue area [50].

Combined with current evidence, our findings indicate that physical activity needs to be at least moderate in intensity to influence body weight and levels of adiposity. Additionally, preservation of total skeletal muscle mass with a significant reduction in total and abdominal adipose tissue measured by magnetic resonance imaging was detected independent of exercise amount or intensity in 103 adults with abdominal obesity [51]. Similarly, our data also failed to detect differences in lean body mass across physical activity categories.

MedDiet is a dietary pattern that has been praised for its success in promoting weight loss and reducing long-term weight gain if energy restriction exists [52]. However, contradictory findings have been reported between MedDiet adherence and changes in weight or overweight/obesity risk [53,54]. The number of calories of MedDiet components, such as olive oil, whole cereals, and nuts, might explain why we failed to detect differences in obesity indices across MedDiet categories, suggesting that participants in the ILERVAS trial with higher adherence to MedDiet failed to achieve a negative energy balance. In fact, the traditional nutritional pattern that characterizes MedDiet can exert its effect through dissimilar pathophysiological mechanisms not related with weight loss, such as improving the lipid profile, modulating inflammation, enhancing its anti-oxidant properties, and reducing blood pressure and insulin resistance, among others [55,56].

Randomized clinical trials and meta-analyses support that physical activity added to dietary intervention further enhances the amount of weight loss achieved [57]. Contrary to expected, our findings in a large overweight population with moderate cardiovascular risk failed to demonstrate a positive interaction between physical activity practice and MedDiet adherence and adiposity indices. It is well known that lifestyle interventions on diet and exercise promotion are especially effective at reducing weight in those individuals with obesity, whereas participants in the ILERVAS project were especially overweight.

Potential limitations must be highlighted in our study. First, we lack the true percentage and distribution of body fat determined with a gold standard tool. Also, we have not evaluated other obesity indices that include the hip circumference, such as abdominal volume index, body adiposity index, and waist to hip ratio. Therefore, we are not sure whether the inclusion of hip circumference would modify our results. However, the close relationship between obesity indices and adiposity has been well established, and remarkable differences between these were not detected in our large population. Second, as an observational and cross-sectional study, a causal relationship between physical activity practice and adherence to the Mediterranean dietary pattern and excess body adiposity cannot be established. Third, the time of physical activity and food consumption was self-reported by participants. Since the study did not use the triaxial accelerometer or doubly labeled water to accurately determine physical activity level or energy expenditure, neither a weighing dietary record to obtain a detailed dietary pattern, this is an important limitation of the study. Fourth, information about socio-demographic and lifestyle characteristics, which have been related to body composition, was not available from the participants included in the study, as well as information about whether participants performed hypocaloric diets or had taken protein supplements in previous months [58,59]. Finally, results from this study cannot be extrapolated to other populations, since our population comprised of Spanish middle-age individuals that were overweight and at low-to-moderate cardiovascular risk.

In conclusion, our records demonstrate that moderate to vigorous physical activity practice is related to lower obesity indices in a large sample of middle-aged subjects. However, MedDiet showed a slight impact on anthropometric indices. There are also no joint associations between physical activity and MedDiet with lower adiposity measurements. In a world, where most adults are performing less than the recommended intensities of physical activity, our results encourage the inclusion of physical activity as an important lifestyle behavior for regulating body composition and minimizing the comorbidities associated with excess body fat [60].

## Figures and Tables

**Table 1:** Main clinical characteristics and comorbidities of the entire study population according to their physical activity practice.

	Low PA	Moderate PA	Vigorous PA	*p* *	*p* **
Number (%)	4408 (66.0)	1884 (28.2)	380 (5.6)	-	-
Women, *n* (%)	2137 (48.5)	1192 (63.3)	97 (25.5)	<0.001	<0.001
Age (years)	57 (52; 62)	59 (54;64)	54 (50; 60)	<0.001	<0.001
Current or former smoker, *n* (%)	2704 (61.3)	992 (52.7)	220 (57.0)	0.186	0.062
Hypertension, *n* (%)	1764 (40.0)	778 (41.3)	125 (32.9)	0.006	0.002
Systolic blood pressure (mmHg)	131 (120; 142)	129 (119; 142)	127 (117; 137)	0.091	0.768
Diastolic blood pressure (mmHg)	82 (75; 88)	81 (75; 87)	81 (75; 87)	0.146	1.000
Antihypertensive drugs, *n* (%)	1464 (33.2)	660 (35.0)	101 (26.6)	0.008	0.001
Dyslipidemia, *n* (%)	2276 (51.6)	1031 (54.7)	203 (53.4)	0.503	0.642
Total cholesterol ≥200 mg/dL, *n* (%)	2385 (54.1)	1086 (57.6)	196 (51.6)	0.343	0.030
Lipid-lowering agents, *n* (%)	797 (18.1)	381 (20.2)	71 (18.7)	0.770	0.494
Obesity, *n* (%)	1361 (30.9)	561 (29.8)	78 (20.5)	<0.001	<0.001
BMI (kg/m^2^)	28.8 (25.9; 32.2)	28.3 (25.4; 31.6)	27.7 (25.1; 30.2)	<0.001	0.002
Antithrombotic drugs, *n* (%)	138 (3.1)	57 (3.0)	13 (3.4)	0.756	0.684

* High vs. Low PA ** High vs. Moderate PA. Data are expressed as median (interquartile range) or *n* (percentage). PA: Physical activity; BMI: body mass index. Antihypertensive drugs include angiotensin-converting enzyme (ACE) inhibitors, diuretics, angiotensin-II receptor antagonists (ARA II), beta-blockers, calcium antagonists, and other antihypertensives. Lipid-lowering treatments involve statins, fibrates, ezetimibe, and omega-3 fatty acids. Antithrombotic drugs include anticoagulants and antiplatelets.

**Table 2:** Main clinical characteristics and comorbidities of the study population according to their adherence to the Mediterranean diet.

	Low MedDiet	Moderate MedDiet	High MedDiet	*p* *	*p* **
Number (%)	1076 (16.1)	5054 (75.7)	542 (8.1)	-	-
Women, *n* (%)	419 (38.9)	2695 (53.3)	213 (57.6)	<0.001	0.060
Age (years)	55 (51; 60)	58 (53; 63)	58 (54; 64)	<0.001	0.240
Current or former smoker, *n* (%)	763 (70.9)	2896 (57.3)	257 (47.4)	<0.001	<0.001
Hypertension, *n* (%)	397 (36.9)	2036 (40.3)	234 (43.2)	0.015	0.193
Systolic blood pressure (mmHg)	129 (119; 142)	130 (120; 142)	129 (119; 137)	0.257	0.257
Diastolic blood pressure (mmHg)	82 (76; 88)	81 (75; 88)	80 (74;87)	0.003	0.093
Antihypertensives, *n* (%)	333 (30.9)	1710 (33.8)	182 (33.6)	0.284	0.905
Dyslipidemia, *n* (%)	512 (47.6)	2686 (53.1)	312 (57.6)	<0.001	0.050
Total cholesterol ≥200 mg/dL, *n* (%)	572 (53.2)	2781 (55.0)	314 (57.9)	0.069	0.196
Lipid-lowering agents, *n* (%)	187 (17.4)	958 (19.0)	104 (19.2)	0.371	0.895
Obesity, *n* (%)	213 (29.0)	3515 (30.5)	149 (27.5)	0.527	0.154
BMI (kg/m^2^)	28.7 (25.5; 32.0)	28.7 (25.8; 32.0)	27.9 (25.2; 31.4)	0.041	0.001
Antithrombotic drugs, *n* (%)	35 (3.3)	158 (3.1)	15 (2.8)	0.594	0.647

* High vs. Low adherence to Mediterranean diet ** High vs. Moderate adherence to Mediterranean diet. Data are expressed as median [interquartile range] or n (percentage). MedDiet: Mediterranean diet; BMI: body mass index. Antihypertensive drugs include angiotensin-converting enzyme (ACE) inhibitors, diuretics, angiotensin-II receptor antagonists (ARA II), beta-blockers, calcium antagonists, and other antihypertensives. Lipid-lowering treatments involve statins, fibrates, ezetimibe, and omega-3 fatty acids. Antithrombotic drugs include anticoagulants and antiplatelets.

**Table 3:** Obesity indices in male and female participants according to their physical activity practice.

**MALE SUBJECTS**	**Low PA**	**Moderate PA**	**Vigorous PA**	*p* *	*p* **
*n*	2270	692	283	-	-
Total adiposity					
BMI (Kg/m^2^)	29.0 (26.4; 32.0)	28.6 (26.0; 31.3)	27.8 (25.4; 30.2)	<0.001	0.004
CUN-BAE (%)	30.7 (27.3; 34.2)	30.2 (27.0; 33.8)	29.0 (25.8; 32.3)	<0.001	<0.001
Deurenberg (%)	31.4 (28.0; 35.0)	31.0 (27.8; 35.1)	29.3 (26.4; 32.8)	<0.001	<0.001
Central adiposity					
Waist circumference (cm)	102 (96; 110)	101 (94; 108)	98 (90; 104)	<0.001	<0.001
Conicity index	1.34 (1.30; 1.38)	1.33 (1.29; 1.38)	1.30 (1.25; 1.30)	<0.001	<0.001
Waist to height ratio	0.60 (0.56; 0.64)	0.59 (0.55; 0.64)	0.57 (0.53; 0.61)	<0.001	<0.001
Bonora (cm^2^)	196 (158; 247)	190 (145; 234)	171 (120; 209)	<0.001	<0.001
A body shape index	0.083	0.083	0.081	<0.001	<0.001
	(0.081; 0.085)	(0.081; 0.085)	(0.079; 0.084)		
Body roundness index	5.34 (4.54; 6.34)	5.25 (4.36; 6.24)	4.63 (3.92; 5.61)	<0.001	<0.001
Neck circumference (cm)	41.0 (39.0; 43.0)	40.5 (39.0; 42.5)	40.0 (38.0; 42.0)	<0.001	0.048
Lean body mass					
Hume (kg)	56.4 (52.5; 60.4)	55.4 (51.6; 59.2)	55.6 (52.2; 59.6)	0.197	1
**FEMALE SUBJECTS**					
*n*	2137	1192	97	-	-
Total adiposity					
BMI (Kg/m^2^)	28.7 (25.3; 32.5)	28.1 (25.2; 31.9)	27.0 (24.6; 30.1)	0.006	0.081
CUN-BAE (%)	42.5 (38.4; 46.4)	42.0 (38.4; 45.9)	40.4 (37.2; 43.6)	0.004	0.036
Deurenberg (%)	32.1 (27.7; 36.8)	31.6 (27.6; 36.1)	29.4 (26.6; 33.4)	0.003	0.018
Central adiposity					
Waist circumference (cm)	100 (92; 108)	98 (90; 106)	96 (89; 103)	0.008	0.312
Conicity index	1.36 (1.31; 1.41)	1.35 (1.30; 1.40)	1.33 (1.30; 1.39)	0.04	0.641
Waist to height ratio	0.64 (0.58; 0.69)	0.62 (0.58; 0.68)	0.60 (0.57; 0.65)	<0.001	0.046
Bonora (cm^2^)	188 (153; 224)	183 (150; 218)	170 (140; 202)	0.004	0.051
A body shape index	0.085 (0.082; 0.088)	0.084 (0.081; 0.087)	0.084 (0.081; 0.087)	0.69	1
Body roundness index	6.17 (5.02; 7.46)	5.92 (4.87; 7.20)	5.45 (4.62; 6.60)	<0.001	0.046
Neck circumference (cm)	35.0 (33.5; 37.0)	35.0 (33.0; 36.5)	34.5 (33.0; 36.0)	0.043	0.586
Lean body mass					
Hume (kg)	43.3 (39.8; 46.8)	42.5 (39.3; 46.2)	42.4 (39.9; 46.1)	1	1

* Low vs. High PA ** Moderate vs. High PA. Data are expressed as a median [interquartile range]. PA: physical activity; BMI: body mass index; CUN-BAE: Clinica Universidad de Navarra-Body Adiposity Estimator.

**Table 4:** Results of the obesity indices in male and female participants according to their adherence to the Mediterranean diet.

**MALE SUBJECTS**	**Low MedDiet**	**Moderate MedDiet**	**High MedDiet**	*p* *	*p* **
*n*	657	2359	230	-	-
Total adiposity					
BMI (Kg/m^2^)	28.7 (25.9; 31.6)	28.9 (26.4; 31.7)	28.1 (25.8; 31.5)	1	0.101
CUN-BAE (%)	30.1 (26.6; 33.7)	30.6 (27.3; 34.0)	29.5 (26.6; 33.8)	1	0.138
Deurenberg (%)	30.5 (27.2; 34.5)	31.3 (28.0; 34.9)	30.2 (27.3; 34.9)	1	0.246
Central adiposity					
Waist circumference (cm)	101 (94; 109)	102 (96; 109)	100 (94; 107)	0.057	0.057
Conicity index	1.33 (1.29; 1.38)	1.33 (1.30; 1.38)	1.32 (1.28; 1.36)	0.164	0.015
Waist to height ratio	0.59 (0.55; 0.64)	0.60 (0.56; 0.64)	0.58 (0.55; 0.63)	0.592	0.023
Bonora (cm^2^)	190 (145; 241)	196 (158; 241)	183 (145; 228)	0.06	0.06
A body shape index	0.083	0.083	0.082	0.101	0.036
	(0.081; 0.085)	(0.081; 0.085)	(0.080; 0.085)		
Body roundness index	5.22 (4.26; 6.23)	5.29 (4.49; 6.26)	4.97 (4.31; 6.13)	0.592	0.023
Neck circumference (cm)	40.5 (39.0; 42.5)	41.0 (39.0; 43.0)	40.5 (39.0; 43.0)	0.176	0.176
Lean body mass					
Hume (kg)	56.5 (52.3; 60.1)	56.0 (52.1; 60.2)	55.9 (52.6; 59.9)	0.594	0.594
**FEMALE SUBJECTS**					
*n*	419	2695	312	-	-
Total adiposity					
BMI (Kg/m^2^)	28.5 (24.9; 32.8)	28.5 (25.3; 32.3)	27.8 (24.9; 31.1)	0.077	0.02
CUN-BAE (%)	42.3 (37.9; 46.6)	42.4 (38.5; 46.3)	41.7 (37.8; 45.3)	0.285	0.041
Deurenberg (%)	31.7 (26.9; 36.8)	32.0 (27.8; 36.6)	31.3 (27.1; 35.6)	0.07	0.07
Central adiposity					
Waist circumference (cm)	99 (90; 107)	99 (91; 107)	98 (90; 105)	0.087	0.087
Conicity index	1.35 (1.31; 1.40)	1.36 (1.30; 1.41)	1.36 (1.30; 1.40)	0.465	0.465
Waist to height ratio	0.63 (0.58; 0.68)	0.63 (0.58; 0.69)	0.62 (0.57; 0.68)	0.441	0.024
Bonora (cm^2^)	182 (144; 218)	187 (153; 222)	182 (151; 215)	0.082	0.082
A body shape index	0.084 (0.081; 0.087)	0.085 (0.081; 0.088)	0.085 (0.082; 0.088)	0.156	0.156
Body roundness index	6.14 (4.84; 7.24)	6.07 (5.00; 7.38)	5.73 (4.79; 7.14)	0.441	0.024
Neck circumference (cm)	35.0 (33.5; 37.0)	35.0 (33.5; 37.0)	34.5 (33.0; 36.5)	0.015	0.149
Lean body mass					
Hume (kg)	43.7 (39.8; 47.0)	42.9 (39.6; 46.5)	41.9 (39.5; 46.3)	0.095	0.095

* Low vs. High PA ** Moderate vs. High PA. Data are expressed as a median (interquartile range). MedDiet: Mediterranean diet; BMI: body mass index; CUN-BAE: Clinica Universidad de Navarra-Body Adiposity Estimator.

**Table 5:** Disjoin and join effects of physical activity and the Mediterranean diet in obesity indices according to gender.

**MALE SUBJECTS**	**Physical Activity**	**Mediterranean Diet**	**Physical Activity and Mediterranean Diet**
**Total adiposity**			
BMI (kg/m^2^)	0.003	0.774	0.509
CUN-BAE (%)	<0.001	0.424	0.518
Deurenberg (%)	<0.001	0.237	0.606
Central adiposity			
Waist circumference (cm)	<0.001	0.697	0.538
Conicity index	<0.001	0.328	0.889
Waist to height ratio	<0.001	0.295	0.77
Bonora (cm^2^)	<0.001	0.721	0.584
A body shape index	<0.001	0.405	0.917
Body roundness index	<0.001	0.287	0.783
Neck circumference (cm)	0.079	0.887	0.369
Lean body mass			
Hume (kg)	0.383	0.436	0.216
**FEMALE SUBJECTS**			
Total adiposity			
BMI (kg/m^2^)	0.061	0.435	0.808
CUN-BAE (%)	0.067	0.385	0.835
Deurenberg (%)	0.045	0.304	0.837
Central adiposity			
Waist circumference (cm)	0.01	0.161	0.335
Conicity index	0.023	0.051	0.243
Waist to height ratio	0.008	0.106	0.436
Bonora (cm^2^)	0.007	0.044	0.442
A body shape index	0.198	0.073	0.503
Body roundness index	0.008	0.125	0.461
Neck circumference (cm)	0.192	0.508	0.612
Lean body mass			
Hume (kg)	0.39	0.295	0.511

Two-way ANOVA was used for all *p*-values. BMI: body mass index; CUN-BAE: Clinica Universidad de Navarra-Body Adiposity Estimator.

## Supplementary Materials

The following are available online at https://www.mdpi.com/2072-6643/11/6/1359/s1, Figure S1: Flow chart of the study population.

## Appendix A

Author List and Affiliations of ILERVAS Project collaborators:

Ferran Barbé ^1,2^, José Manuel Valdivielso ^3^, Gloria Arqué ^4^, Silvia Barril ^1,2^, Ana Vena ^4^, Pere Godoy ^5,6^, Mariona Jové ^7^, Montserrat Martínez-Alonso ^8^, Eva Miquel ^9,10^, Marta Ortega-Bravo ^5,9^, Manel Portero-Otin ^7^, Serafín Cambray ^3^, Gerard Torres ^1,2^ and Eva Castro ^3^

1 Respiratory Department, Arnau de Vilanova-Santa María University Hospital, Translational Research in Respiratory Medicine, IRBLleida, University of Lleida, Lleida, Spain.
2 Centro de Investigación Biomédica en Red de Enfermedades Respiratorias (CIBERES), Instituto de Salud Carlos III (ISCIII), Madrid, Spain.
3 Vascular and Renal Translational Research Group, IRBLleida, RedinRen-ISCIII, Lleida, Spain.
4 Stroke Unit. University Hospital Arnau de Vilanova, Clinical Neurosciences Group, IRBLleida, University of Lleida, Lleida, Spain.
5 Applied Epidemiology Research Group, IRBLleida, Agència de Salut Pública de Catalunya, Lleida, Spain.
6 Centro de Investigación Biomédica en Red de Epidemiología y Salud Pública (CIBERESP), Instituto de Salud Carlos III (ISCIII).
7 Department of Experimental Medicina, IRBLleida, University of Lleida, Lleida, Spain.
8 Systems Biology and Statistical Methods for Biomedical Research Group, Biostatistics Unit, IRBLleida, Universitat de Lleida, Lleida, Spain.
9 Unitat de Suport a la Recerca Lleida, Fundació Institut Universitari per a la recerca a l’Atenció Primària de Salut Jordi Gol i Gurina (IDIAPJGol), Barcelona, Spain.
