# Supplementary figures and images for: Dissimilar Impact of a Mediterranean Diet and Physical Activity on Anthropometric Indices: A Cross-Sectional Study from the ILERVAS Project
Source: Nutrients. 2019 Jun 17;11(6):1359. doi: 10.3390/nu11061359 (PMC6627626; doi:10.3390/nu11061359)

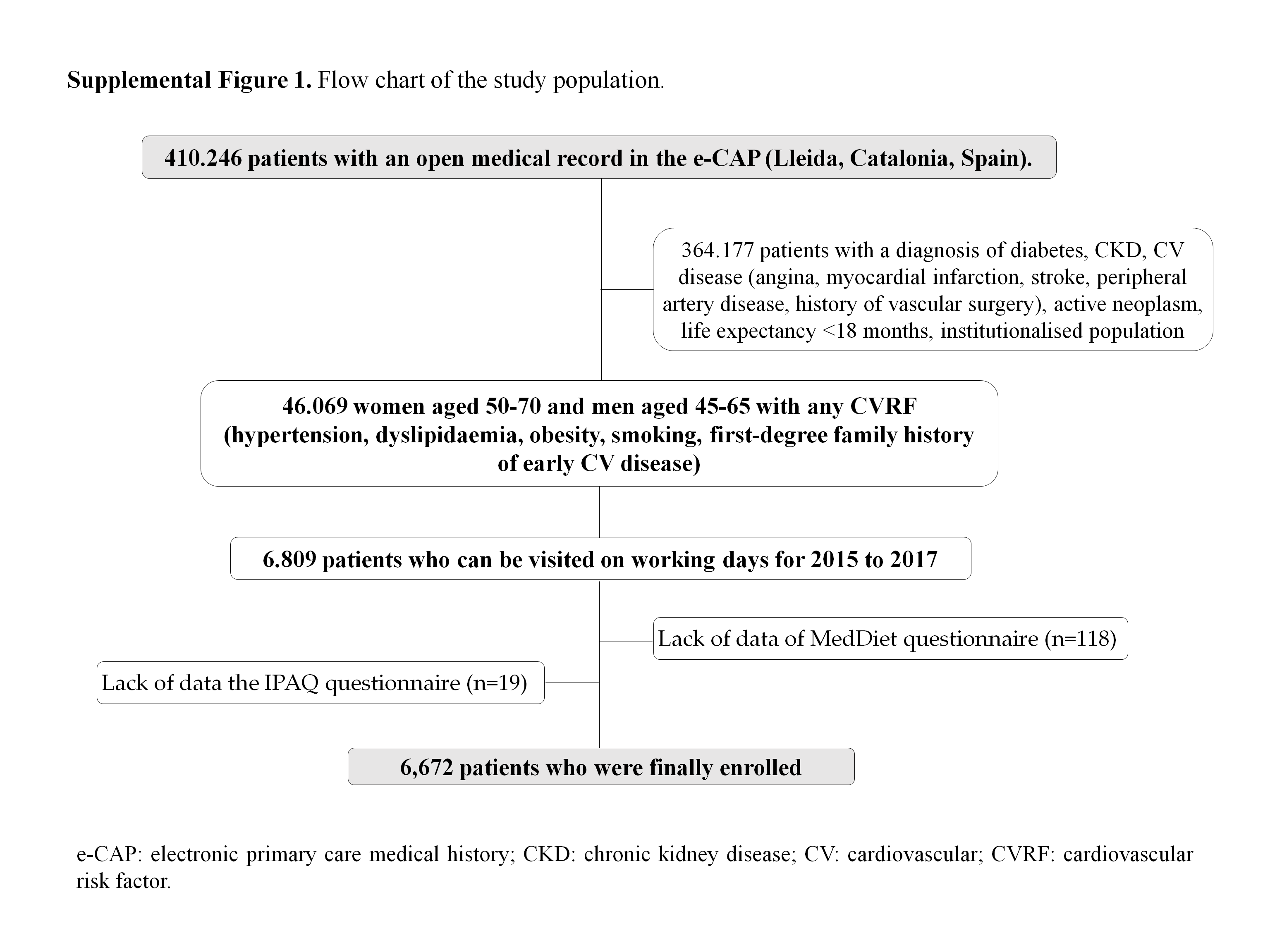

Supplement: Supplementary file 1 [file nutrients-11-01359-s001.zip › nutrients-515577-supplementary.tif]
